# A novel balloon-assisted technique to secure visceral catheterization during a chimney endovascular repair of a ruptured abdominal aortic aneurysm in a centenarian patient

**DOI:** 10.1590/1677-5449.202300182

**Published:** 2023-11-13

**Authors:** Francisco Leonardo Galastri, Leonardo Guedes Moreira Valle, Marcela Juliano Silva Cunha, Bruno Pagnin Schmid, Rodrigo Gobbo Garcia, David Salomão Lewi, Breno Boueri Affonso, Felipe Nasser

**Affiliations:** 1 Hospital Israelita Albert Einstein – HIAE, São Paulo, SP, Brasil.

**Keywords:** endoleak, abdominal aortic aneurysm, endovascular procedure, aneurysm, ruptured, aged, 80 and over, case report, endoleak, aneurisma de aorta abdominal, procedimentos endovasculares, aneurisma roto, idoso de 80 anos ou mais

## Abstract

A 100-year-old male patient was admitted with a ruptured abdominal aortic aneurysm due to type IA endoleak. Given the proximity of the ruptured site to the superior mesenteric artery (SMA) and renal arteries, a ChEVAR was indicated. Catheterization of the target visceral vessels was a challenging procedural step because of an intensely tortuous thoracic aorta. This hostile aortic anatomy also inhibited exchange for a super stiff guide-wire and selective cannulation with the diagnostic catheter was repeatedly lost when guidewire exchange was attempted. To overcome this issue, a 5 x 40 mm balloon catheter was placed 3cm into the target arteries. The balloon was then inflated below the nominal pressure limit enabling safe exchange for a super stiff guidewire and placement of three 90-cm long 7Fr guiding sheaths. The procedure was thus safely performed with deployment of an aortic extension and the bridging stents.

## Resumo

Um paciente de 100 anos foi diagnosticado com um aneurisma de aorta abdominal roto por um endoleak 1A. Pela proximidade do ponto de ruptura com a artéria mesentérica superior (AMS) e as artérias renais, um ChEVAR foi indicado. A cateterização das artérias-alvo foi um passo desafiador pela intensa tortuosidade da aorta torácica. Essa anatomia aórtica hostil também impediu a troca por um fio-guia extra-rígido, e a cateterização seletiva foi repetidamente perdida quando a troca de fio-guia foi tentada. Para superar essa dificuldade, um cateter balão 5mm x 40mm foi posicionado nas artérias-alvo. O balão foi, então, insuflado abaixo da pressão nominal, permitindo uma troca segura do fio-guia por um fio-guia extra-rígido e o posicionamento de três bainhas longas 7Frx 90cm. Assim, o procedimento foi executado de forma segura, com o implante de uma extensão aórtica e dos stents recobertos.

## INTRODUCTION

A ruptured abdominal aortic aneurysm is a catastrophic event, especially in the elderly population, with a significant peri-operative mortality rate.^1,2^

Endovascular repair using the chimney technique (ChEVAR) emerges as a valuable option in these emergent scenarios.^3^ ChEVAR is also useful as a “bail-out” procedure for accidentally overstented aortic branches, for treatment of distal arch aneurysms, and in elective scenarios for patients who are poor surgical candidates for open repair or unsuitable for fenestrated endovascular repair (F-EVAR).^4^

However, catheterization of the visceral vessels is one of the most challenging procedural steps involved in this technique, requiring advanced wire and catheter handling skills, which usually prolongs procedure duration and radiation exposure and can be associated with serious complications such as arterial dissection and arterial perforation.^3-6^

This report aims to describe a simple and helpful balloon-assisted maneuver to secure visceral catheterization of the target vessels during ChEVAR.

## PART I – CLINICAL SITUATION

This report received local ethical committee approval (Consolidated opinion: 5553161, CAAE number: 60593822300000071).

A 100-year-old male Caucasian patient was admitted to the Emergency Department of a tertiary hospital in January 2022 with sudden intense abdominal pain and hemodynamic collapse.

Physical examination found the patient oriented with heart rate at 120 bpm, low blood pressure (90/50 mmHg), and a distended abdomen with a large pulsatile abdominal mass.

His medical history was notable for previous treatment of a 6.5cm abdominal aortic aneurysm (AAA) with endovascular aortic aneurysm repair (EVAR) using a bifurcated stent graft in 2015 and two reinterventions due to a type IIB endoleak. This EVAR was performed at a different institution and no additional information regarding the procedure was available.

The first reintervention was performed in January 2020 using direct percutaneous aneurysm sac puncture and injection of an ethylene–vinyl alcohol copolymer liquid embolic agent (Onyx 18, Medtronic-Covidien, Irvine, CA).

Despite all efforts, the patient’s aneurysm sac continued to expand (maximum diameter: 11 cm) and so a second reintervention was performed in June 2021 using a percutaneous approach and superselective angiography to administer a precipitating hydrophobic injectable liquid (PHIL, MicroVention, Tustin, California) injection.

## PART II – WHAT WAS DONE

Given the patient’s clinical presentation and medical history, a diagnosis of a ruptured abdominal aneurysm due to a possible endoleak was inferred. No preoperative CT was ordered. The institution’s protocol was therefore activated, triggering the blood bank, the interventional radiology team, anesthesiologists, and hybrid room preparation simultaneously.

The patient was promptly conducted to the interventional department with a 20-minute emergency department to hybrid room time.

A permissive hypotension strategy was implemented to target a systolic blood pressure of less than 90 mmHg.^7^ Intravenous cefazolin was administered prior to puncture.

The procedure was headed by 3 interventional radiologists (10 years in practice) assisted by 3 interventional radiology fellows.

**Figure 1:**
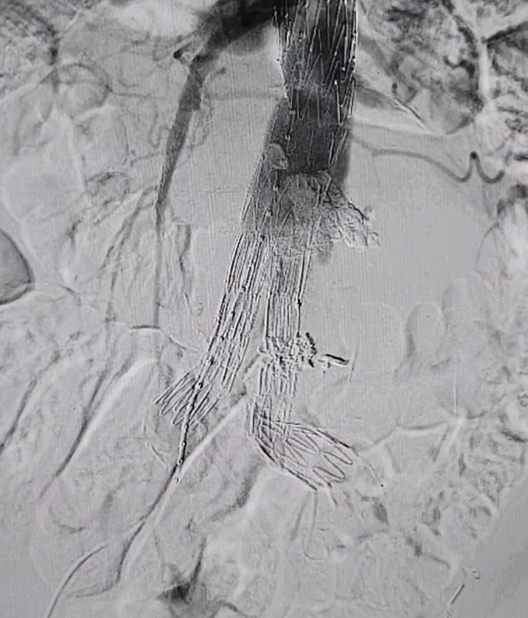
Initial angiography showing contrast extravasation at the proximal aortic neck (Type IA endoleak).

Under local anesthesia, using retrograde percutaneous access via the right common femoral (5 Fr), a 5 Fr pigtail catheter (Cordis Corporation®, Miami lakes, FL, USA) was positioned at the T12 level. Initial angiography showed a type IA endoleak and a ruptured aneurysm. (Figure 1).

Given the proximity of the rupture site to the superior mesenteric artery (SMA) and renal arteries, the patient’s poor clinical status, and the urgent need for rapid sealing, the chimney technique (ChEVAR) using bridging stents to the SMA and to the renal arteries was indicated.^3,8^

Ultrasound-guided bilateral access to the brachial arteries (one vascular access to the right brachial artery and 2 vascular accesses to the left brachial artery) was obtained and a left femoral access was then secured utilizing a preclose technique with pre-placement of 2 Perclose ProGlide (Abbott Vascular, California, United States) closure devices.^9^

Catheterization of the visceral vessels (SMA and renal arteries) from the brachial access was then performed using a 0.035-inch stiff hydrophilic coated guidewire (Terumo, Tokyo, Japan) and a 4Fr Vertebral catheter (Cordis Corporation, Miami lakes, FL, USA).

Catheterization of the target vessels was a challenging procedural step due to an intensely tortuous thoracic aorta.

This hostile aortic anatomy also inhibited exchange for a 0.035-inch Amplatz Super Stiff guidewire (Boston Scientific CorporationNatick, MA) and selective cannulation with the vertebral catheter was repeatedly lost when guidewire exchange was attempted.

To overcome this issue and secure visceral catheterization during guidewire exchange, the vertebral catheter was retrieved, and a 5 x 40 mm Passeo-35 balloon catheter (Biotronik AG, Buelach, Switzerland) was placed 3cm into the target arteries.

The balloon was then inflated below the nominal pressure limit (5 atm) enabling safe exchange for a super stiff guidewire and subsequent easy placement of three 90-cm long hydrophilic-coated 7Fr guiding sheaths (Flexor Ansel, Cook Medical, Bloomington, IN, USA).^10-15^

Following the standard fashion, using a Lunderquist Extra Stiff Wire Guide (Cook Medical, Bloomington, IN, USA), a 20Fr introducer sheath (Medtronic, Minneapolis, United-States) was placed in the left femoral artery and a 36 x 49 mm aortic extension (Endurant II, Medtronic, Minneapolis, United States) was deployed to cover the thoracic portion of the type IA endoleak zone aiming for 30% oversizing. The aortic graft was molded using a compliant balloon (Reliant balloon catheter, Medtronic, Minneapolis, United States).

A balloon expandable stent (VBX 9x79 mm, Gore, Flagstaff, AZ, United-States) was used to bridge the SMA, whereas one self-expandable stent (Viabahn 7x100 mm, Gore, Flagstaff, AZ, United States) was necessary for each renal artery.

Intraoperative complications included a type 3 endoleak in the left iliac limb treated with deployment of a 16 x 24 x 124 mm iliac extension (Endurant II, Medtronic, Minneapolis, United States) and a left renal hemorrhage of an inferior segment, resolved with superselective catheterization using a Renegade microcatheter (Boston Scientific Corporation Boston Scientific, Natick, MA) and injection of a 2 mL mixture of N-butyl-2-cyanoacrylate (Histoacryl glue, B Braun, Sheffield, UK) and ethiodized oil (Lipiodol,Guerbet, Solihull, UK) (glue: lipiodol = 1:2).

**Figure 2:**
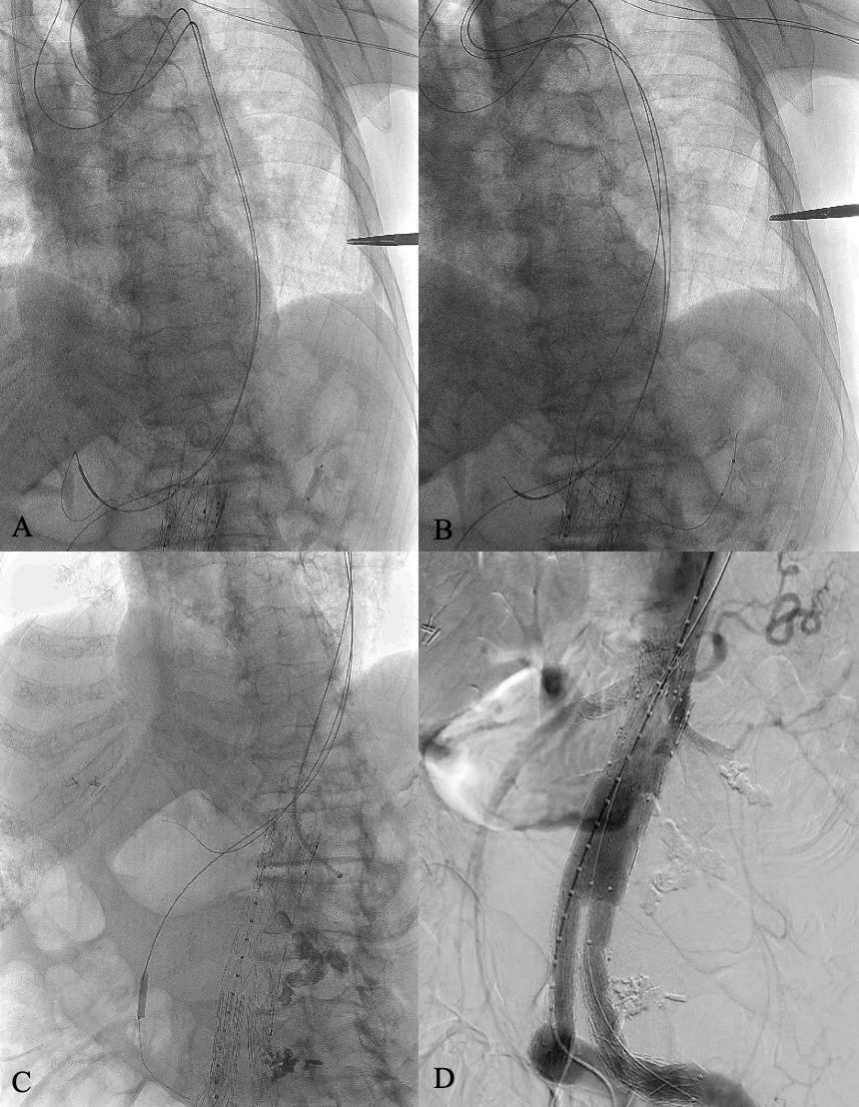
The balloon-assisted technique. **(A)** A balloon is positioned into the left renal artery; **(B)** The balloon is gently inflated below the nominal pressure limit (5 atm); **(C)** Guidewire exchange for a super stiff guidewire can be safely performed and a guiding sheath can be easily delivered into the target artery; **(D)** The completion angiography showing complete endoleak resolution, patency of all target arteries, and no blush.

The completion angiography at the end of the procedure showed no residual endoleaks and patency of all target arteries (Figure 2).

The suture-mediated Perclose ProGlide (Abbott Vascular, California, United States) closure device was used for all vascular accesses to achieve hemostasis and reduce vascular complications.

During the follow-up period, the patient underwent contrast-enhanced ultrasound (CEUS) and computed tomography angiography (CTA) and both imaging exams demonstrated patent stent grafts without any endoleaks.

Despite the technical success, he died on postoperative day 59 due to respiratory complications.

## DISCUSSION

This report presents a simple technique that can help to secure visceral catheterization of target vessels during ChEVAR of ruptured abdominal aortic aneurysms.

There are several previously described endovascular maneuvers that can facilitate vessel catheterization during aneurysm aortic repair.^16,17^

However, the challenging condition in the present case was not limited to catheterization of the target vessels, but also involved maintenance of the diagnostic catheter during guidewire exchange, due to the intense aortic tortuosity. This is a commonly faced situation during endovascular repair and demands creative technical solutions.

The evidence of this case, the technique has potential for reducing fluoroscopic time, but it is fair to say that this still needs validation.

Moreover, the technique can reduce catheter manipulation in the visceral branches’ ostia, which can possibly attenuate the risk of vessel perforation and dissection, as noted in this patient.

Additional advantages include its simple execution and the low-cost endovascular devices needed, usually widely available in aortic centers.

Finally, another key point to discuss from the present technical report is the feasibility of performing ChEVAR in a centenarian patient.

Several groups have reported encouraging results describing EVAR in octagenarians and nonagenarians, but the literature regarding centenarians is limited.^18^ This case opens the debate about use of ChEVAR in carefully selected geriatric patients, who have historically been turned down for abdominal aortic aneurysm repair.

The novel balloon-assisted technique is a simple and helpful maneuver to secure visceral catheterization of target vessels during ChEVAR of ruptured abdominal aortic aneurysms, especially in patients with intense aortic tortuosity.
